# Integrating Traditional Medicine and Healing into the Ghanaian Mainstream Health System: Voices From Within

**DOI:** 10.1177/10497323211008849

**Published:** 2021-05-13

**Authors:** Abukari Kwame

**Affiliations:** 1 Centre for Sami Studies, The Arctic University of Norway, Tromsø, Norway; 2 University of Saskatchewan, Saskatoon, Canada

**Keywords:** traditional medicine, integrative health care system, medical worldviews, power politics, Ghana, interpretive ethnography

## Abstract

In this study, I employed interpretive ethnographic qualitative design to explore perceptions of and proposals from traditional healers, biomedical practitioners, and health care consumers regarding integrating traditional medicine and healing in Ghana. Data were gathered through focus groups, in-depth individual interviews, and qualitative questionnaires and analyzed thematically. The results revealed positive attitudes toward integrating traditional medicine in Ghana and a discursive discourse of power relations. The power imbalance between biomedical and traditional practitioners regarding what integrative models to adopt is sanctioned by formal education and institutional structure. As a result, multiple approaches for integration were made, including patient co-referrals, collaborations between biomedical and traditional medical practitioners, and creating a unit for traditional medicine and healers at the outpatients’ department for patients to choose either biomedicine or traditional medicine. Incorporating aspects of traditional healing in the training of biomedical practitioners and creating a space for knowledge sharing were also proposed. These integrative models reflected the distinctive interests of healers and biomedical practitioners. Considering these findings, I recommended policy options for consideration toward achieving an integrative health care system in Ghana.

## Background

Traditional medicine and healing (TMH) practices are used globally, making significant contributions to meet the health care needs of populations in developing countries and some developed countries, including the United Kingdom, Canada, the United States, and Australia (Hilbers & Lewis, 2013; Hussain & Malik, 2013; Payyappallimana, 2010). TMH is effective for mental health illnesses (Akol et al., 2018; Gureje et al., 2015; Kpobi et al., 2019; Musyimi et al., 2016), which is often a challenge for clinicians, according to traditional healers specialized in mental health illnesses (Akol et al., 2018). Moreover, the World Health Organization (WHO, 2002) estimates that about 60% of the global population uses herbal medicine to treat their illnesses and up to 80% of the African population depends on traditional medicine for their primary health care needs. Most traditional medical practices are compatible with their users’ cultural values, beliefs, and worldviews regarding the meanings of health, illness, and healing (Barimah, 2013). This holistic nature of traditional medical therapies is what makes them favorable among many rural and low-income populations.

In Ghana, where the study that informs this article occurred, TMH is an accessible, affordable, available, and effective health care system that provides care services to over 70% of the population (Asante & Avornyo, 2013; Aziato & Antwi, 2016; Barimah, 2013; Barimah & Akotia, 2015; Krah et al., 2018). Furthermore, Ofosu-Amaah (2005) argues that in Ghana, Mali, Nigeria, and Zambia, herbal medicine is the first option for treating 60% of children with a malaria-induced high fever.

Countries like China, Japan, Singapore, India, the Republic of Korea, and Hong Kong have integrated TMH into their mainstream health care systems (Hussain & Malik, 2013; Nimoh, 2014). In these countries, patients can access it exclusively or together with other biomedical forms of therapies. These countries’ success stories make it imperative that we explore how TMH can be integrated into the Ghanaian formal health care system, given the contributions of TMH to the health care needs of the Ghanaian population and the disparities in the distribution of health facilities and personnel between rural and urban Ghana.

The governments of Ghana and the Ministry of Health have created policy options and institutional structures toward integrating TMH in Ghana. The Center for Scientific Research into Plant Medicine (CSRPM), established in 1975 at Mampong-Akwapim, through Dr. Oku Ampofo, works to promote the development of herbal medicine. The Center has botanical gardens, conducts scientific research into plant medicine, and produces high-quality herbal medicine (Asante & Avornyo, 2013). Working with the Noguchi Memorial Institute for Medical Research and other commercial organizations, the center disseminates research findings and technical information on herbal medicine in Ghana. Furthermore, in 1991, the Ministry of Health established the Traditional and Alternative Medicine Directorate to provide a well-defined and recognized complementary health system based on excellence in traditional and alternative medical knowledge and to coordinate and monitor all traditional medical practices in Ghana (Ministry of Health, 2003, 2005). Within the same period, the Ghana Federation of Traditional Medicine Practitioners (GHAFTRAM) was created as a mother association for all regional and local healer associations (Asante & Avornyo, 2013; Nimoh, 2014). These developments were to organize and promote the professional development of traditional healers.

Besides, the Government of Ghana in 2000 passed the Traditional Medicine Practice Act (Act 575), which mandated the establishment of a traditional medicine council. This Council is responsible for registering and licensing traditional medical practitioners, regulating traditional medical practices, and the preparation and sale of herbs and herbal medicines (Government of Ghana, 2000). However, the Council’s secretariat was set up in 2004, and the Council itself was constituted and inaugurated on April 9, 2010. Moreover, the integration of herbal medicine has been piloted in 17 hospitals in Ghana, including the Kumasi South hospital, with a few reported successes and challenges (Agyei-Baffour et al., 2017; Boateng et al., 2016). Despite these developments, TMH is yet to be fully integrated into the formal health care system in Ghana, and traditional healers operate on the fringes of the formal health care system. Thus, although these structures have been put in place to get traditional healers organized and trained, to develop herbal medicine, and to promote an integrative medical system in Ghana ultimately, several factors suggest that Ghana does not operate an integrative health care system yet, as far as TMH is concerned.

First, many biomedical practitioners see traditional practitioners’ work as illegal; hence, they do not refer patients to them, which constitutes a perceptual issue. Second, patients are often harassed or abused by some physicians when they use traditional medicine before accessing biomedicine in the hospital. Third, TMH services are not covered by the National Health Insurance (Barimah, 2013). Fourth, there is no systemic education in TMH where prospective healers are formally trained, although a Bachelor of Science degree program in Herbal Medicine is being run by the Kwame Nkrumah University of Science and Technology (Asante & Avornyo, 2013; Aziato & Antwi, 2016). Finally, Asante and Avornyo (2013, p. 263) observed that there are “only a few institutions whose traditional medical practices are officially recognized by government,” which is the CSRPM. This assertion suggests that many traditional medical practitioners outside the various research institutional settings are unrecognized. Based on the above, Ghana can best be described as running a tolerant or inclusive health care system (Barimah, 2013; Vasconi & Owoahene-Acheampong, 2010).

In this article, I explore perceptions and approaches toward integrating TMH in Ghana by examining and interpreting proposals made by traditional healers, biomedical practitioners, and health care consumers. Thus, two questions are explored: What are the perceptions of different actors toward the integration of TMH in Ghana, and how do these actors think TMH can be integrated?

## Types of Medical Systems and Models of Integrating Health Care Systems

Various factors have been noted in the literature concerning integrating TMH into national health care systems in different countries. National health policy and legislation, formal education and training of practitioners, and scientific rationality influence how medical systems are integrated. In this section, I present the different medical systems identified in the literature and the models or approaches used in integrating medical systems.

The WHO (2002) has identified three common ways that countries adopt to make TMH part of their mainstream health care systems. These are integrative, inclusive, and tolerant health care systems.

### Integrative Health Care Systems

According to the WHO (2002), integrative national health care systems^1^ recognize TMH in their national health and drug policies. Traditional healers and medical products are registered and made available in public and private hospitals, and traditional medicines and services are covered by national health insurance. There is significant and ongoing research into TMH practices, education, and training (Kayne, 2010). Integrative health care systems are multidisciplinary and engage interprofessional collaboration between mainstream and alternative medical services (Wiese et al., 2010). Also, there is mutual respect between orthodox and alternative medicine practitioners, and patients’ health care needs are mostly satisfied. China, Vietnam, the Republic of Korea, Singapore, and the Democratic People’s Republic of Korea are the only countries with integrative medical systems (Chang & Basnyat, 2015; Vasconi & Owoahene-Acheampong, 2010; WHO, 2002).

### Inclusive Health Care Systems

In inclusive medical systems, TMH is only formally recognized as a medical system but is not fully covered in the national health care policies. Moreover, inclusive health care systems do not integrate traditional healers’ training into the educational system (Vasconi & Owoahene-Acheampong, 2010; WHO, 2002). Traditional medicine is rarely included in health insurance programs, and there is minimal regulation of the traditional practitioners and their products. Many countries with inclusive systems aim to achieve integrative health care in the future. Developing countries such as Mali, Nigeria, and Equatorial Guinea and developed countries, such as Canada and the United Kingdom, have inclusive medical systems where traditional and complementary medicines and practices are allowed (WHO, 2002, 2019).

### Tolerant Health Care Systems

In these medical systems, only certain practices of TMH are tolerated by the country’s national laws. Orthodox or allophonic medicine dominates in the tolerant medical systems, mostly found in developing countries, including Guinea Bissau, Niger, Bangladesh, Samoa, and Bahamas (WHO, 2002, 2019). In some developed countries, such as the United States, “there is no national plan for integrating T&CM [traditional and complementary medicine] into mainstream health service delivery” although traditional and complementary medical services and practices (e.g., acupuncture) are tolerated (Kielczynska et al., 2014; WHO, 2019, p. 101).

### Parallel Medical Systems

Countries can also adopt parallel medical systems whereby orthodox and traditional medicine are developed and operated separately within the national health care system (Barimah, 2013; Kayne, 2010). It has been shown that India and some Southeast Asian countries practice parallel medical systems (Barimah, 2013; Kayne, 2010). With this system, both medical systems are recognized by law, developed, and allowed to operate separately (Barimah, 2013). Nonetheless, less is known about how practitioners of the different medical systems in parallel health care systems relate or how they perceive each other’s medical practices.

The literature has also identified a few models of integrating medical systems, which may align with some of the types of medical systems presented above (see Asante & Avornyo, 2013). One of the models that countries can use to combine different medical systems is what Isola (2013) called the *institutionalization approach*. By institutionalization, Isola (2013) noted that centers of scientific research into plant medicine are established, and extensive gardens with herbal plants are developed. A research hospital for traditional medicine and university training programs are created to train traditional medical practitioners. Although institutionalization can be an approach to integrating traditional and orthodox medicines, Isola (2013) did not make it clear whether the educational training component argued for is part of the training of biomedical practitioners, or they are meant to produced trained traditional practitioners with exclusive knowledge in TMH.

*Incorporation*/*co-optation* is another model. With this model, there is a selective inclusion of elements of traditional and alternative medicine into a comprehensive treatment plan alongside mainstream diagnostic and treatment practices within hospital programs, where primary physicians are in control (Shuval & Mizrachi, 2004; Shuval et al., 2012; Wiese et al., 2010). Thus, alternative medical practitioners could work in health care facilities under the directives of primary care physicians. This can be a form of institutional integration where traditional healers and biomedical practitioners work in the same institutional setup under biomedical practitioners’ supervision. This model exists in Australia (Wiese et al., 2010) and Israel (Shuval et al., 2012; Shuval & Mizrachi, 2004).

### Pluralization model

With this model, Wiese et al. (2010) observed that health care consumers access the medical system that suits their care needs. Thus, care consumers maintain autonomy while preserving the integrity of the treatment systems involved. The pluralization model is considered patient-centered and allows integration to revolve around health care consumers’ choices (Wiese et al., 2010).

#### Theoretical framework

Positioning Theory, conceived as the discursive production of selves in conversation and narrative (Davies & Harré, 1990; Deppermann, 2013; Harré et al., 2009), was employed in this study. Scholars of Positioning Theory argue that in narratives and conversational exchanges, storylines, social acts, and positions are discursively claimed. Storylines are created to define actors and their relationships in the sequence of actions and events. Social acts are performed, and positions, as moral claims tied to the storylines, are reproduced, contested, and negotiated in the social acts based on the duties and rights the actors claim for themselves (Davies & Harré, 1990).

Deppermann observed that positioning embodied a Faucaudian notion of “subject positions” in the sense that “discourses position subjects in terms of status, power, legitimate knowledge and practices they are allowed to and ought to perform” (Foucault, 1969, cited in Deppermann, 2013, p. 2). Researchers interrogate how people position themselves and others, events, processes, and actions in narratives and social interaction with this theory. Positioning Theory was used as an analytic tool to examine how traditional healers, biomedical practitioners, and health care consumers position one another, the medical systems, and practices in their narratives. What claims of duties and rights and self-identities are produced in discourses concerning the integration of TMH in Ghana.

## Method

### Study Design

This study adopted an interpretive ethnographic qualitative research design (Gehart & Lyle, 2001; Yagi & Kleinberg, 2011) to explore the perspectives, perceptions, experiences, and proposals of participants regarding the integration of TMH into the Ghanaian health care system. Through interpretive ethnography, researchers examine participants’ experiences and their sense-making process as members of a particular cultural group (Yagi & Kleinberg, 2011). How the broader social context shapes the meanings and interpretations people make and the researcher’s understanding and interpretation of participants’ experiences are crucial in interpretive ethnography. I chose this research design to explore participants’ perspectives about how TMH and healers could be integrated and what it means to adopt a particular approach.

### Study Setting

The study was conducted in Northern Ghana among the Dagomba, who constitute the largest ethnic group in the region and are primarily subsistence farmers. Data were collected in and around Yendi, the traditional capital of Dagbon, which is located on the Eastern enclave of the Northern Region. A significant part of Dagbon is rural, with poor road networks and thin distribution of formal health care services. I selected this region because TMH represents a crucial health care system for many inhabitants of this region.

### Participants

Participants for the study were traditional healers, health care service consumers, and biomedical health care practitioners. Policymakers were not included in this study because I was interested in the grassroots participants’ perspectives—people whose work and lives are affected by health policy. I needed to gather their views and experiences to inform the policy proposals in this study to influence evidence-based practice. Purposive sampling was used to recruit participants based on their knowledge of the topic and willingness to participate in the study. Participants were invited through word of mouth, and those who gave their voluntary informed consent were contacted and interviewed. Ten traditional healers, 10 biomedical practitioners, and 14 health care service consumers participated in the study.

All the healers gave their consent to participate by thumbprinting the consent forms, and interview dates were fixed. With the healers’ help, health care consumers were recruited for focus groups in two communities, following the same consent procedure. Regarding the biomedical practitioners, I contacted them individually, explained the study’s purpose to them, and asked for their consent to participate after gaining approval and access from the hospital management. Eighteen participants signed the consent forms and took the qualitative questionnaires, of which 17 were returned after 3 weeks. Ten questionnaires were fully completed and used in the study.

### Data Collection

The data reported in this study were part of a more extensive study on TMH in Northern Ghana, as reported in Kwame (2016a, 2016b). Three main objectives were explored in that study (a) to examine the Dagomba traditional medical knowledge and practices, (b) to determine what influences people’s choice for TMH, and (c) to explore perceptions toward and approaches for integrating TMH into Ghana. The data reported in this study concern only the third objective. See Kwame (2016b) for results for the first and second objectives.

I used two separate semi-structured interview guides and a qualitative questionnaire with open-ended questions in the study. The interview guide for healers contained questions that explored their knowledge acquisition process, areas of healing expertise, illness causation, healing practices, and how clients perceive their work. Other questions explored their perceptions toward integrating TMH and how that could be done. The interview guide for the focus groups explored health care consumers’ knowledge of illness causation, medical systems they access and why, their perceptions about TMH practices, its integration into the main health care system, and how. The qualitative questionnaire contained open-ended questions with spaces for participants to write their responses for each question. Questions explored biomedical practitioners’ knowledge about traditional medicine, perceptions toward traditional medicine and healers, categories of healers biomedical practitioners would like to work with, and why, and illnesses that they thought traditional medicine and healers could handle well and why, issues around patient referral, their perceptions of integrating TMH in Ghana, and how. See the Online Appendix for the interview guides for objective (c).

Fieldwork for the study was conducted from June to August in 2015. Twenty face-to-face in-depth individual interviews were held with healers at their homes. Two focused groups were conducted with health care consumers (one group had seven males and the other seven females), and 10 completed qualitative questions from biomedical practitioners.

Detailed ethnographic observations of healing practices were conducted over 3 weeks with healers, which gave me a firsthand impression of their healing practices, herbal medicinal preparation, and the illnesses they heal. I also kept reflexive fieldnotes throughout the process. In-depth individual interviews followed these observations, and each healer was interviewed twice on separate days—individual interviews lasting an average of 1 hour and each focus group 1½ hours. I also had informal conversations with the healers, which deepened and challenged the rapport between us, as reported in Kwame (2017). All interviews and focus groups were conducted in Dagbani, the native language of both the participants and researcher.

### Data Analysis and Rigor

Interviews and focus groups were audio-recorded with the consent of participants. I listened to each audio recording several times, transcribed them verbatim in Dagbani, and translated them into English but retained crucial Dagbani expressions during data transcription. I coded and reflected on initial interview transcripts before I conducted subsequent interviews. Transcribed data were read out to participants during follow-up meetings for validation and correction, where necessary. Data were manually coded employing Smith et al.’s (1999) approaches to allow for interpretive analysis later. Thus, I closely read data transcripts several times to immerse myself in the data and made interpretive comments to summarize and show associations or connections with previous data. Keywords and phrases and how participants made sense of the phenomenon were noted on one margin of the transcript and emergent thematic codes and my initial interpretations on the other, as Smith et al. (1999) advised.

All data sources (i.e., interviews, focus groups, and observation notes) and participants’ transcripts were compared for similarities, differences, and contradictions. As Tobin and Begley (2004, p. 393) argued, triangulation serves as a means of “offering a deeper and more comprehensive picture” of the phenomenon under investigation. I organized all the codes generated into themes following Braun and Clarke (2006) by first employing inductive thematic analysis to discover themes based on participants’ perspectives. The semantic content of themes was briefly described and moved further to the interpretive level (Braun & Clarke, 2006), where I analyzed their significance and broader meanings and implications on how TMH can be integrated in Ghana.

Two research assistants helped to cross-check the emergent themes to ensure these were reflected in the data. For instance, the theme of “differences in medical worldviews,” especially between healers and biomedical practitioners, was significant and led to power issues, which then influenced the integrative approaches the practitioners proposed for TMH.

### Ethics and Consent Process

Ethics approval was granted by the Norwegian Social Science Data Services (NSD; Ref: 43718 / 3 / MSI) and the hospital in Yendi. After obtaining ethics approval from my university, I made a formal application to the hospital management, which was approved and access granted. All local and cultural ethical protocols were observed, and participants gave their voluntary consent for participation. During the data analysis and report writing, participants’ identifying information was removed and replaced with serial codes and numbers (although serial codes are not used in this article).

## Findings

### Study Participants

Thirty-four participants, consisting of 10 healers, 10 biomedical practitioners, and 14 health care consumers, participated in this study. Among the healers, two were females and eight were males. The healers had an age range of 35 to 85 years, with a mean age of 59 years. Seven of them had no formal education, two had primary, and one had tertiary-level education. Nine of the healers were Muslims, whereas one was a Traditional religious practitioner. The majority of them combined healing with farming or trading, and three practiced healing as their primary occupation. On average, the healers had 27 years of healing experience. The biomedical practitioners consisted of eight males and two females. Four were Christians and six were Muslims, and they all had tertiary levels of education. The biomedical practitioners had an age range of 25 to 60 years, with a mean age of 43 years. On average, they had 19 years of practice experience. The care consumers were seven men and seven women. All of them were Muslims. The men had an age range of 30 to 75 years, with a mean age of 57 years, whereas the women had an age range of 25 to 89 years, with a mean age of 50 years. All the men were farmers, whereas the women had varying occupations, ranging from farming, trading, and food vending to primary school cook. Only one of the care consumers had primary education; the rest had no formal education.

### Differences in Medical Worldviews as a Challenge for Integrating TMH

Different philosophies, beliefs, perspectives, and realities were expressed among the participants about TMH and its integration in Ghana. These ideologies and worldviews were mostly related to healing practices, belief systems, and social relationships.

One healer (an herbalist) indicated that working with the formal health care system (embodied as “the hospital”) will be challenging due to differences in medical beliefs. He remarked,

I won’t go to the hospital to heal. Yes, in Dagbanli, we are not used to taking your secrets somewhere. When you do that, the medicine will die. Even as a healer, when an illness is caused by sorcery/witchcraft, and you go ahead to heal that, you are likely to be attacked or killed.

A spiritualist also stated that working with the formal health care system means that one could be paid for his or her services. However, he beliefs that this could violate his medical philosophies, as noted below:

The reason why I won’t like to work in the hospital is what I told you earlier about. They’ll use money to convince/trick me (*n yohim ma*). But that weakens medicine; the medicine I’ve is for me to save lives but not make money.

Another healer (a snake bite specialist) alluded to the differences in medical beliefs and practices in the following quote:

Can I leave home and stay (work) in the hospital at my age? I won’t like to work that way, because I’m very old now. Besides, as we heal people, we moistly boil mɔʔu (medicine) and do other things. Can we boil mɔʔu in the hospital? Or can I ask nurses to run errands for a patient or me?

In this quote, the participant has positioned himself as “aged and weak.” He has also positioned his medical ideology as conflicting with that of the mainstream health care system. As a result, working in the hospital will be challenging for his medical practices. The *Self* is positioned as lacking the power to ask the hospital staff to carry out healing tasks for him, which avows power differences between healers and biomedical practitioners, a theme I will discuss later.

Among the biomedical practitioners, a participant noted that TMH is different from biomedicine, as presented below:

As the name implies, traditional healers use traditional approaches which mostly have no scientific basis. I’d prefer working with only healers who have had formal education and training. That’ll ensure that clients are safely treated. Healers should have minimum accreditation, licensing standards.

According to this participant, there are limited scientific approaches to what traditional healers do in Ghana. Hence, healers are positioned as lacking scientific knowledge to heal illnesses. They are assumed to have inadequate diagnosing procedures and attach so much spirituality to their work.

During the first focus group, a health care consumer whose relative’s son had a fractured leg and was receiving treatment in a hospital lamented the situation. She maintained that if the boy was not taken to traditional healers, the hospital might amputate his leg. Another health care consumer remarked that “the boy should be sent to Pansiya for traditional healing; it’s effective.” In the second focus group, another health care consumer argued that traditional healing has practices that cannot be performed in the hospital:

Traditional healing is actually different. There is that secrecy (*ashili*) in it. Some of their practices cannot be done openly. They may have to prepare mɔʔu (m pɔ mɔʔu) for the patient to bath with it. But how can they take mɔʔu to the hospital? Another issue is that you may be asked to provide a hen or a cock for healers to prepare the medicine. These are some things that make it secretive.

These discourses highlighted the differences in medical philosophy and practices between the traditional and biomedical systems, which these participants feel can affect TMH and healers’ integration into the mainstream health care system. Other incompatible traditional medical practices noted were ritual practices, spiritual healing, and preparation of herbal medicine.

A few biomedical practitioners indicated that “healers are poorly trained and cause more harm than good,” and others argued that “healers operate in secrecy and spiritualism and are not willing to share their knowledge about healing practices.” Another biomedical practitioner felt that traditional medicines are “unhygienic,” prepared under poor surroundings, have “poor dosage,” and “no expiration dates.” Other biomedical practitioners believed that most healers are less educated, lack basic training and knowledge about the illnesses they treat or the medicine they use, have substandard illness-diagnosing procedures, and are not certified. For these reasons, it might be hard for them to work with traditional healers.

### Power Dynamics Between Healers and Biomedical Practitioners

A dominant discourse that was recurrent among participants regarding integrating TMH and healers into the formal health care system was the ideology of power, medical knowledge positioning, and having control over medical practices and patients. One healer narrated this about power dynamics:

I usually go to the hospital to see patients when their relatives call on me. And sometimes, the nurses in the ward prevent me from seeing the patient; the doctors/nurses don’t usually allow me. Sometimes when they do, it’s only on persistence, then I will be allowed in. Some of the doctors may allow me in because they want me to help them treat the patient.

In the above narrative, the act of nurses preventing healers from seeing patients at the hospital already highlights the feeling most healers have about working with the formal health care system and with biomedical practitioners. The unequal power relations between healers and biomedical practitioners that position the latter as dominant can influence the working relationship between the two groups of medical practitioners, especially in an institutionalized integrative health care system. Thus, many healers noted that institutional integration of TMH that allows healers and biomedical practitioners to work in the same wards could be problematic as they might not have full control over patients, visitors, and healing practices.

Many care consumers acknowledged the discursive discourse of power when a health care consumer stated that “it’s the doctors who have to agree that they can work with healers before the healers can work in the hospital.” Through this statement, the participant acknowledged the privileged positions—institutionally and professionally—that doctors enjoy, thereby positioning healers as less powerful and requiring medical doctors’ permission to work in the hospital. Another care consumer stated that when a healer gives a patient *tisablim* (traditional medicine) before taking them to the hospital, nurses and doctors get annoyed:

When you use traditional medicine for your illness before seeing the doctor, the hospital people don’t want that. So, since doctors in the hospital don’t usually accept this, a healer would not want to work in the hospital. In most cases, we have to take the patient out of the hospital ward before giving them *tisablim*.

A biomedical practitioner also asserted that “if traditional healers *comply with our regulations*, we’ll be happy working with them.” By owning the regulations (rather the medical system), the participant acknowledges and privileges biomedical practitioners’ power.

### Attitudes Toward Integration

Many healers, care consumers, and some biomedical practitioners were optimistic about integrative health care in Ghana. One healer acknowledged that integrating TMH into the formal health care system could offer patients the opportunity to disclose the use of TMH when asked by biomedical practitioners. He noted that patients are usually insulted at the hospital when nurses and doctors notice that a patient has been using TMH for specific illnesses. The healer added that nurses and doctors often accuse patients who use TMH of being stingy and do not want to spend money for better treatment. A fracture healer also indicated that it would be useful to integrated TMH. He noted, “We want to work with the mainstream health care system. Today, things have changed from how they used to be” because nowadays they use specific tools and medicines from the orthodox medical field in traditional healing.

Furthermore, many care consumers stated that it would be nice to have TMH integrated into the main health care system. One care consumer stated,

Yes. It will be good to have healers as part of the main health system. Because they may take a patient to the hospital and the physicians won’t be able to treat him or her, but a healer may treat the illness.

Another care consumer asserted that healers could even earn salaries for working with the formal health care system as a form of reward for providing care services.

Although paying healers salary can contribute to their income base when they are integrated into the formal health care system, it could also conflict with some healers’ personal ideologies, especially healers in Dagbon that believe that “money kills the medicine,” so traditional medicine should not be sold. For these healers, receiving a salary for providing healing services could be interpreted as selling their medicine.

Among the biomedical practitioners, one of them agreed that integrating healers and TMH would be great because some healers, such as herbalists, can effectively treat many illnesses; also, most orthodox medicines came from herbs. Another biomedical practitioner stated that “if healers become part of the formal health system, it’ll be good since the workload on regular [biomedical] staff will reduce.” This participant implied that the integration of TMH and healers into the formal health care system is welcome insofar as it reduces the workload on biomedical practitioners.

However, other biomedical practitioners expressed ambivalent attitudes toward integrating TMH. First, two biomedical practitioners indicated that they preferred orthodox medicine and so will not recommend traditional medicine or some healers to their clients because traditional healing may be ineffective. Yet, one of them acknowledged that he lacks knowledge about TMH or what healers do and that the “lack of knowledge about traditional medicine among many biomedical practitioners could post serious challenges to integration.” The other practitioner indicated that he would only recommend traditional healing if biomedicine fails for some illnesses. Both participants said bonesetters could effectively handle bone-setting or simple fractures.

These ambivalent attitudes among some biomedical practitioners about TMH do not only prevent them from exploring what healers do but could also undermine efforts toward achieving integrative health care in Ghana. Thus, if an institutional mode of integration is achieved and healers and biomedical practitioners have to work in the same health care facility, it may be challenging. Moreover, as hinted earlier, the discursive discourse of power and medical knowledge appeared very influential in how biomedical practitioners perceive healers and their practices, which can hinder institutional integration.

### Medical Innovations and Ways to Integrate TMH in Ghana

Participants noted some innovative medical practices and several other approaches that could enhance TMH and healers’ integration into the Ghanaian formal health care system.

#### Innovative medical practices

Some medical practices were found to be innovations in both medical systems. Regarding the traditional medical system, one innovative medical approach found was utilizing orthodox medical tools among traditional healers and developing local collaborations with biomedical practitioners. A bonesetter/fracture specialist noted,

We use X-ray photos, bandages, and orthodox pain killers in our work. The X-ray photos help us to assess the exact location of a fracture and its severity. If there are cuts, these can be stitched by nurses and wounds dressed. Sometimes we ask the nurses we work with to come and give our patients water, blood, or injections.

Another innovation was developing local collaborations with some biomedical practitioners, as hinted in the above quote. The two traditional bone specialists revealed that they have local partnerships with some biomedical practitioners in a hospital in Yendi. Some of these local collaborations are implemented through either patient co-referral or calling upon the biomedical practitioner to administer blood or water to patients under the healers’ care. For example, a healer who is both an herbalist and spiritualist stated that “Sometimes patients in the hospital are told to seek home treatment for their illness. That’s when their relatives contact us; the nurses in the hospital who know our work can just direct the patient to us.” Another healer, who heals jinn possession, said that a few nurses working in the hospital usually refer patients to him. Thus, for these healers, co-referral of patients and collaboration as integrative models are favored.

Within the formal health care system, one innovative medical practice was noted. This practice involves a situation where biomedical practitioners with traditional medical knowledge from their families combine that with their professional work. A health care consumer observed this practice as narrated below:

Yes, it’ll be nice for biomedical and traditional practitioners to work together. Even in Southern Ghana, it’s already like that. When you go to Duayaw-Nkwanta hospital, the doctor combines his professional work with local fracture healing practices. The fracture healing is his family tradition, so he adds that to his medical career.

A biomedical practitioner revealed a similar practice in the quote below:

I’ll feel comfortable working with healers. In the 1970s, a doctor in this hospital used to treat many patients with traditional knowledge. Even recently, one of our medical assistants incorporates traditional healing in his professional work.

Practices such as this can influence other biomedical practitioners to rethink traditional medicine and their attitudes toward it. It can also encourage traditional healers and biomedical practitioners to work together within one health care facility.

#### Strategies for integrating TMH in Ghana

Many proposals were made concerning how TMH can be integrated into the formal health care system. A bone and fracture specialist said, “I prefer that patients are sent to me, because healing in Dagbon needs to have some secrecy. We can both send patients to each other.” Another healer (an herbalist) suggested co-referral of patients in this narrative:

I’d like to work with hospitals. But you see, *kom kam zori mɨ nɨ dɨ soli* (a native proverb translated as: “different water runs according to their courses”). So, I’ll prefer that patients are referred to me. When I was living around Tamale, I was contacted with this idea, but I decline. I do keep patients here. So, patients can be sent to me here.

A health care consumer proposed a collaborative arrangement. The participant indicated,

The man I talked about earlier goes to the hospital to heal. Since healers can’t just go to the hospital and stay there, they should remain in their homes. The hospital should contact them when an illness requires their attention. The hospital and the healer should have plans so that they will both know how to work together.

Another care consumer added, “Like Afa Yisifu (name of a healer), the doctor knows about him. Illnesses that the doctor doesn’t understand, they take the patient to him.” The participant implies that some hospitals/clinics in Ghana already have working relationships (local links) with healers through informal arrangements.

Many biomedical practitioners recommended formal institutionalization of TMH or aspects of it. For instance, two biomedical practitioners recommended including aspects of TMH in the training of physicians. One of them stated that

Traditional medicine should be integrated into the formal training of physicians. This will give we the biomedical practitioners a fair knowledge about traditional medicine to recommend it to our clients.

With this proposal, the participant presupposes that only biomedical practitioners can sanction the integration of TMH based on the amount of knowledge they have about it. Another biomedical practitioner remarked that

Calculated attempts should be made to integrate healers into the formal healthcare system, . . . but healers will need basic training on how to identify and prioritize sicknesses. They must be certificated and provided continuous professional development.

This participant acknowledges that formal training and certificating of traditional healers are crucial to integrating them into Ghana’s mainstream health care system. Other proposals included setting up a unit in the hospital for traditional healers, where patients are offered the choice of traditional or orthodox medicine at the outpatients’ department (OPD), as noted by one biomedical practitioner below:

Set up a unit for traditional medicine and healers. Offer [patients] the choice to go traditional or orthodox medicine at the outpatients’ department. [Also], there is a need for further studies into the activities of healers. Introduce traditional healing in the medical curriculum for training health personnel. Anyway, people still prefer to go to the herbalist, so healers should be integrated into the formal health system.

These proposals support the complete professionalization and institutionalization of TMH, and although laudable, implementing these may come with other challenges.

Some biomedical practitioners made other alternative proposals. For instance, a biomedical practitioner suggested, “I think the way to include healers should be by inviting them to look at patients and make recommendations.” Another biomedical practitioner remarked that “whether we like it or not, TMH has come to stay; so, there should be a platform for healers and biomedical practitioners to share ideas concerning health care delivery in Ghana.” These biomedical practitioners supported active collaboration and knowledge sharing between them and healers. They believed that creating a shared space to exchange ideas, offer training services, and learn traditional beliefs and practices would be essential for traditional and biomedical practitioners.

## Discussion

Integrating TMH and healers into the formal health care system in Ghana has been a concern for many actors—healers, health care consumers, biomedical practitioners, and policymakers. Integrating TMH will not only encourage the use and development of herbal medicine in Ghana, but it will also widen health care services provided across the country. Nonetheless, the problem requires a holistic examination.

First, there are conflicting attitudes toward the inclusion of healers into the mainstream health care system. Many healers and health care consumers have positive attitudes about integration compared with biomedical practitioners. Many biomedical practitioners argued that traditional healers are less effective and could cause more harm than good. Others maintained that healers use spiritualism and secrecy as a cover-up to claim medical knowledge. These perceptions among biomedical practitioners could be influenced by their lack of information and knowledge about healers and what they do, thereby confirming previous findings (Aziato & Antwi, 2016; Krah et al., 2018). For example, Kielczynska et al. (2014) observed that physicians in a U.S. hospital had little knowledge about acupuncture practices and were hostile to acupuncturists until they began to access acupuncture care services. Several studies have reported physicians’ negative attitudes toward healers in Ghana (Asante & Avornyo, 2013; Gyasi et al., 2011, 2016), Nigeria (Isola, 2013), Cameroon (Wamba & Groleau, 2012), and elsewhere (Chang & Basnyat, 2015; Hall et al., 2018; see also Helman, 2007). Even in settings where healers or alternative practitioners are incorporated in the health care system, they are controlled by physicians and excluded from certain practices and medical subfields (Shuval & Mizrachi, 2004).

The second issue revolves around concerns over traditional medicines’ efficacy and safety, as reported in several other studies (see Aziato & Antwi, 2016; Barimah, 2013; Carrie et al., 2015; Gyasi et al., 2011). Traditional medicines are perceived to have low quality and safety with no expiry dates, poor dosages, are prepared under poor environmental conditions, and hence positioned as substandard and problematic. These perceptions make many biomedical practitioners feel reluctant to recommend TMH for patients. These assumptions about traditional medicine and practices can constrain efforts toward integrating TMH in Ghana. Nonetheless, public education, collaborative knowledge sharing, sustained trust and relationships, and basic training practices among traditional and biomedical practitioners can mediate and inform more positive perceptions about TMH (Krah et al., 2018).

Based on the perceptions found in this study, I argue that many biomedical practitioners in Ghana will prefer the subordination of healers under their directives in hospitals if an institutional model of integration is achieved. This integrative model will offer biomedical practitioners the continual enjoyment of power, prestige, and status in the health care setting. Such a model is already being adopted in Australia (Wiese et al., 2010).

Furthermore, a discursive discourse of power and control was revealed in this study. The lack of formal education among most healers will position them as illiterates and subordinated under biomedical practitioners’ direct supervision in an institutionalized integrative system. The real or imagined powers that healers and care consumers see biomedical practitioners to possess can be argued to emanate from the political and institutionalized structures of formal education and manifested in the hospital’s discursive space, thereby positioning biomedical practitioners as more powerful and knowledgeable than healers. Studies have reported this relational power imbalance between traditional and biomedical practitioners in Ghana and elsewhere (Chang & Basnyat, 2015; Hampshire & Owusu, 2013; Marsland, 2007; Shuval & Mizrachi, 2004; Wamba & Groleau, 2012; Wiese et al., 2010). For instance, Marsland (2007) reported that in Tanzania, biomedical and missionary medics often see traditional healers as backward and ignorant, so they do not refer patients to healers. Similarly, van der Watt et al. (2017) found that perceptions of superiority among biomedical practitioners were noted. Recognizing the power difference and illness epistemologies would explain why almost all healers in this study preferred co-referral of patients and collaboration with biomedical practitioners as the preferred model for integrating TMH in Ghana.

From the above, a concept of “power politics” can be gleaned from traditional and biomedical practitioners’ perspectives, where each party recognizes the importance of power and control in their discursive spaces of medical practice. Healers want to maintain power and control over their field by proposing co-referral arrangements and local collaborations. In contrast, biomedical practitioners prefer the incorporation of TMH and healers under the directives of the biomedical field, its standards, and practitioners. Due to these power dynamics, any policy on integration that Ghana or other countries wish to adopt must examine the “power politics” objectively and its transformative contextual imperatives within the country’s cultural, historical, and environmental landscapes.

Furthermore, strategic positioning and incorporating biomedical tools and practices into traditional healing seem a growing phenomenon among many traditional healers. Orthodox medicines, X-ray photos, wound dressing equipment, establishing local and informal links/relationships with biomedical practitioners and hospitals for services, such as blood and water transfusion, injections, and so on, were some innovations found within the traditional medical system. Hampshire and Owusu (2013) have reported similar innovative practices. These innovations can have positive impacts on the integration of TMH in Ghana. These innovations suggest that healers are willing to upgrade their practices to make TMH much more attractive. The innovative practices further imply that appropriate measures to enhance the safety, quality, and hygiene of traditional medicines are being implemented by traditional healers, thus positioning them as progressive and dynamic.

Patient co-referrals between the biomedical and the traditional medical systems are reported in the literature (Asante & Avornyo, 2013; Gyasi et al., 2011; Hampshire & Owusu, 2013; Wamba & Groleau, 2012; Wiese et al., 2010). Although traditional healers refer patients to the hospital than biomedical practitioners do to the traditional system, a well-coordinated co-referral system can be an effective way of integrating TMH in Ghana. Recognizing that some healers and TMH are effective will position orthodox medicine, the biomedical field, and the traditional system as having their own limitations in providing health care. Therefore, a potential integrative model for TMH in Ghana is patient co-referral and must be considered seriously in health policy. Moreover, patients are already using all the medical systems, suggesting that medical pluralization and consumer engendered models of integration, as proposed in the literature (see Asante & Avornyo, 2013; Wiese et al., 2010, for details), are already operating in Ghana. Thus, I argue that medical pluralism is an inherent health care consumer behavior whenever different medical systems exist in a society. Health care consumers will continue seeking healing for their illnesses from diverse medical systems available to them, whether there is a formal policy recognizing all the medical systems, their practitioners, and services or not. As a result, I do not consider this as an integrative model.

Biomedical practitioners mostly made proposals for formal institutionalization, standardization, and professionalization of TMH and healers. Although these proposals are significant, policymakers and implementers must remember that biomedicine and TMH have different and sometimes conflicting philosophies about reality, illness causation, and healing practices. Particularly, including spirituality and ritual practices in the formal training of physicians may be challenging. Moreover, some biomedical practitioners saw herbal medicine as representing the entirety of TMH. Although developing TMH could be beneficial in the long run, emphasizing only institutionalization and professionalization models might compromise the dynamism in the plural health care system in Ghana. Issues of spiritual healing and ritual practices can disappear in the process of formal institutionalization of TMH.

## Toward Integrating TMH in Ghana: Policy and Practical Considerations

I offer the following long- and short-term proposals toward the integration of TMH in Ghana. First, there needs to be public awareness about the legality status of TMH. Over 70% of the Ghanaian population relies on TMH for their health care needs (Asante & Avornyo, 2013; Gyasi et al., 2016; Hampshire & Owusu, 2013). Therefore, the public must know the legal status of TMH to monitor the activities of healers and medical products to enhance their quality and safety.

Formal collaboration and patient co-referral arrangements between the health care systems are crucial to strengthening the existing local and informal partnerships. This will create room for knowledge sharing, as practiced elsewhere (Kielczynska et al., 2014; Shuval & Mizrachi, 2004) as many biomedical practitioners do not have adequate knowledge and information about TMH. Such collaborations will enhance the effective management of power and ideological differences between practitioners of both medical systems. Moreover, it will offer traditional healers the opportunity to learn basic hygienic practices relating to herbal medicine preparation, as done elsewhere (see Carrie et al., 2015; Shuval & Mizrachi, 2004). Besides, biomedical practitioners can become culturally sensitive to patients’ lay medical knowledge and beliefs and why they often combine traditional and biomedicine.

Furthermore, traditional healers who already have their clinics, such as Dr. Sahara, Dr. Sammy, and Dr. Nsemka, as reported in Hampshire and Owusu (2013), and Sheikh Salawatia Islamic clinic, can be recognized nationally and supported for research into traditional medical practices. Patients can be formally referred to them from public/private health care facilities for traditional healing.

For the long-term integration strategies, the Ministry of Health, the Ghana Health Service, and the CSRPM must conduct systematic periodic surveys to identify traditional healers in different cultural groups and categorize them according to their specialties (e.g., herbalists, snakebite specialists, bonesetters, spiritualists, Islamic-based healers). The survey should identify how healers may want to be integrated: whether they prefer to work in hospitals with biomedical practitioners, to have local clinics where healers work together, or through established co-referral agreements with hospitals. Where healers and biomedical practitioners have to work in the same care institution, “power politics” must be addressed in such arrangements. For healers whose cultural values, personal belief systems, and worldviews require that they cannot work directly with hospitals, co-referral arrangements will be ideal for them. For instance, most healers in Dagbon believe that selling their medicine can kill its potency (Bierlich, 2007; Krah, 2019; Kwame, 2016b). For a successful nationwide survey, community member participation will be essential in identifying genuine healers in their communities who effectively provide traditional medical services. Both self-identification by healers and community members’ verification will be significant because there are “fake/false healers” (Aziato & Antwi, 2016), traditional medicine sellers, and other groups who may self-identify as healers, just for selfish benefits.

Finally, aspects of traditional healing (cultural beliefs, lay health and healing perspectives, culturally specific illness epistemologies, etc.) can be included in the training modules of biomedical professionals. The existing BSc program in herbal medicine at the Kwame Nkrumah University of Science and Technology is commendable and must be expanded. In addition, research collaboration with healers can be undertaken to conduct clinical trials on healers’ traditional medicines. A comprehensive articulation of traditional knowledge ownership rights, benefit-sharing, and other patent laws on intellectual property rights will be crucial to guide such research and collaboration. It is usually tempting to adopt best practices from other countries when designing policies. Nonetheless, Ghana’s cultural, environmental, and political contexts and discourses must be considered when formulating policies on integrative health care.

A limitation of this study is that the findings cannot be generalized over the entire country. The majority of the healers and care consumers had low educational status, suggesting that different views might be expressed if a sample with many literate participants is used. Despite that, this study has confirmed findings in previous studies and reported novel results.

## Supplemental Material

sj-docx-1-qhr-10.1177_10497323211008849 – Supplemental material for Integrating Traditional Medicine and Healing into the Ghanaian Mainstream Health System: Voices From Within Click here for additional data file. Supplemental material, sj-docx-1-qhr-10.1177_10497323211008849 for Integrating Traditional Medicine and Healing into the Ghanaian Mainstream Health System: Voices From Within by Abukari Kwame in Qualitative Health Research

## Notes

1. In this study, participants initially understood integration as “hospital-based health care,” which according to them was going to be difficult to achieve, especially for healers, due to issues around medical beliefs, philosophies, and practices. However, when I asked them to identify other ways to achieve integration, patient co-referral, collaborative knowledge sharing, healer invitations to attend to specific illnesses in hospitals, and incorporating aspects of TMH into the formal training of biomedical practitioners were offered, thereby broadening the meaning of integration to mean giving formal and legal recognitions to, developing policies, and training opportunities that allow both medical systems to provide health care.
