# Supplementary material for: Integrating Traditional Medicine and Healing into the Ghanaian Mainstream Health System: Voices From Within
Source: Qual Health Res. 2021 May 13;31(10):1847–60. doi: 10.1177/10497323211008849 (PMC8446885; doi:10.1177/10497323211008849)
Supplement: sj-docx-1-qhr-10.1177_10497323211008849 – Supplemental material for Integrating Traditional Medicine and Healing into the Ghanaian Mainstream Health System: Voices From Within [file sj-docx-1-qhr-10.1177_10497323211008849.docx]

# **Appendix: Interview Guides**

## **Semi-structured interview guide for traditional healers**

1. Tell me about yourself and your healing profession.
2. How did you learn to heal?
3. What illnesses do you treat people with?
4. If you are to become part of the formal healthcare system in Ghana,
5. Would you like to work with the healthcare system?
6. Would you like to work with doctors? Why, and Why not?
7. How will you like to work with the formal health system?

Probes:

1. How will you like to be included in the formal healthcare system?
2. How best can you work with the formal healthcare system?
3. What things can facilitate or challenge you from working with the formal healthcare system?

Probes:

1. What do you think can prevent the integration of TMH and healers into the formal healthcare system in Ghana?
2. How can the integration of TMH and healers into the formal healthcare system be made smooth?
3. In what ways will you like to be made part of the formal healthcare system?
4. What perceptions do you think biomedical practitioners have about healers and TMH?
5. How do you feel about the work of biomedical practitioners?

## **Semi-structured interview guide for healthcare consumers**

1. Where do you go for treatment when you are ill?

Probes:

1. Which healthcare system do you access when you are ill?
2. What kinds of illnesses do you send to traditional healers or the hospital?
3. Why do you take some illnesses to the hospital and others to healers?
4. What category of traditional healers do you go to for healing and why?
5. Do you think traditional healers should be made part of the formal health system? Why or why not.
6. What factors can prevent or make difficult the integration of TMH and healers in Ghana?

Probe: What challenges do you foresee regarding integrating TMH and healers in Ghana?

1. What do you propose should be the best ways healers and TMH can be made part of the health system?
2. How should healers and TMH be integrated?
3. How best do you think healers can work with the formal healthcare system?
4. What can facilitate the integration of healers and TMH in Ghana?
5. What difficulties/challenges do you go through to access TMH?
6. What are your experiences regarding combining TMH and biomedicine and how do biomedical professional feel about that?

## **Questions on the qualitative questionnaire for biomedical practitioners**

These questions were used to explore biomedical practitioners’ perceptions and knowledge about traditional medicine and healing, traditional healers, and the integration of TMH and healers in Ghana.

1. Do you find traditional medicine to be effective for some illnesses?
2. What kinds of illnesses do you find traditional medicine to be effective for?
3. Have you ever recommended some of your clients to seek traditional healing for their illnesses?
4. What illness was it, and why?
5. What are some of the reasons that will make you hesitate or reject the use of traditional medicine?
6. Are there aspects of traditional healing that you think should be improved or changed, and why?
7. What benefits you think traditional medicine or healing have?
8. How do you feel about the work of traditional healers?
9. Do you find healers to be effective in treating illnesses, If Yes, how?
10. If No, what are your reasons?
11. Do you know any traditional healer(s) who work in or with this hospital?
12. If Yes, who is the person and what illness(s) does the person heals?
13. If traditional healers were to become part of the health system, how will you feel working with them?
14. Which category of traditional healers will you prefer to work with and why?
15. What are some of the reasons that will make you hesitate or reject treatment from traditional healers?
16. Do you think TMH and healers should be integrated into the formal healthcare system of Ghana? Why or why not?
17. If traditional healers are to become part of the formal health system, what will you propose should be the best ways of integrating TMH and healers in Ghana?
18. What factors can facilitate the successful inclusion of TMH in the formal health system?
19. What factors can impede the integration of TMH and healers in Ghana?
